# 77286 Intravital microscopy in the study of the tumor vasculature of patients with peritoneal carcinomatosis

**DOI:** 10.1017/cts.2021.506

**Published:** 2021-03-30

**Authors:** Emmanuel Gabriel, Minhyung Kim, Daniel Fisher, Catherine Mangum, Kristopher Attwood, Wenyan Ji, Debabrata Mukhopadhyay, Sanjay Bagaria, Matthew Robertson, Tri Dinh, Keith Knutson, Joseph Skitzki, Michael Wallace

**Affiliations:** 1 Mayo Clinic Florida; 2 Roswell Park

## Abstract

ABSTRACT IMPACT: Investigation of tumor-associated blood vessels may serve as an imaging biomarker of response to systemic therapy and cancer outcomes. OBJECTIVES/GOALS: Aberrancies in the tumor microvasculature limit the systemic delivery of anticancer agents, which impedes tumor response. Using human intravital microscopy (HIVM), we hypothesized that HIVM would be feasible in patients with peritoneal carcinomatosis (PC) and generate clinical utility. METHODS/STUDY POPULATION: During cytoreductive surgery with hyperthermic intraperitoneal chemotherapy for PC, HIVM was performed in both tumor and non-tumor areas. The primary outcome was HIVM feasibility to measure vessel characteristics. We secondarily evaluated associations between HIVM vessel characteristics and oncologic outcomes (RECIST response to neoadjuvant therapy and disease-specific survival). RESULTS/ANTICIPATED RESULTS: Thirty patients with PC were enrolled. Nineteen patients (63.3%) received neoadjuvant therapy. HIVM was feasible in all patients. Compared to non-tumor (control) areas, PC areas had a lower density of functional vessels, higher proportion of non-functional vessels, smaller lumenal diameters, and lower blood flow velocity. Qualitative differences in these vessel characteristics were observed among patients who had partial response, stable disease, or progressive disease after receiving neoadjuvant therapy. However, no statistically significant relationships were found between HIVM vessel characteristics and oncologic outcomes. DISCUSSION/SIGNIFICANCE OF FINDINGS: These novel findings comprise the first-in-human, real-time evidence of the microscopic differences between normal and tumor-associated vessels and form the basis for our larger, ongoing clinical trial appropriately powered to determine the clinical utility of HIVM (NCT03823144).

